# Supplementary material for: Dopamine encoding of novelty facilitates efficient uncertainty-driven exploration
Source: PLoS Comput Biol. 2024 Apr 16;20(4):e1011516. doi: 10.1371/journal.pcbi.1011516 (PMC11051659; doi:10.1371/journal.pcbi.1011516)
Supplement: S1 Appendix — (PDF) [file pcbi.1011516.s001.pdf]

S1 Appendix. Cursory analysis of recording data from choice task.

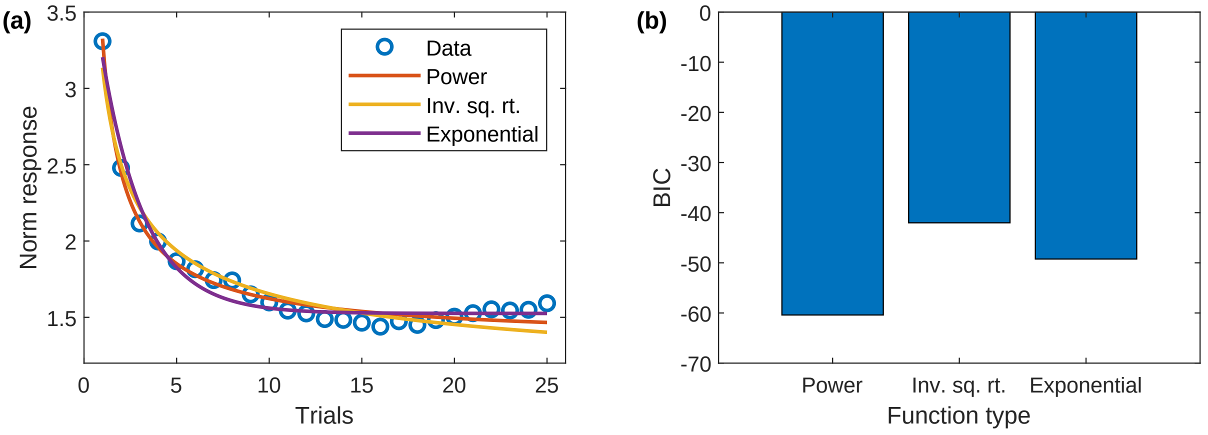

Fig A. Function fitting to data extracted from Figure 6A of [1] (with the average taken from the three curves). **(a)** Normalised neural activity is plotted against number of trials a novel action has been previously taken; three different functions (as described in main text) were fitted to the data points. **(b)** The power function is the best fitting according to the BIC values achieved from function fitting.

In addition to the Pavlovian learning task from [1] described in the main text, the authors also performed an experiment with a two-alternative choice task, during which VTA dopaminergic neurons were recorded. In the recorded phase of the experiment, the animals chose between one familiar option with 50% probability of a high reward and 50% probability of a low reward that they had extensive prior experience with, and one novel option with unknown reward probability. The novel option on each trial had a high reward probability of 25%, 50% or 75% (each reward probability associated with a different visual stimuli as in the Pavlovian learning task). On trials where the novel option was chosen, the early phase activity of recorded dopaminergic neurons show a novelty response pattern similar to that observed for the Pavlovian task regardless of the reward probability of the action. Failing to locate the original dataset from this experiment, we extracted data points from the three curves in Figure 6A of [1], and took the average of all three to produce data points in Fig A(a). We fitted the same functions to these data following the procedure described in the main text for fitting using mean neural activity. The results show that the general power function is still the best fitting function. This cursory analysis suggests that the VTA dopaminergic neurons can still encode novelty of individual actions when there are multiple actions available.

References

1. Lak A, Stauffer WR, Schultz W. Dopamine neurons learn relative chosen value from probabilistic rewards. eLife. 2016;5:e18044. doi:10.7554/eLife.18044.
