# Supplementary material for: Dopamine encoding of novelty facilitates efficient uncertainty-driven exploration
Source: PLoS Comput Biol. 2024 Apr 16;20(4):e1011516. doi: 10.1371/journal.pcbi.1011516 (PMC11051659; doi:10.1371/journal.pcbi.1011516)
Supplement: S1 Table — As described in the main text, we fitted functions to the mean activity of all neurons recorded during the Pavlovian learning task, and also utilised the activity of individual neurons to perform hierarchical model fitting. For fitting using average activity, the best fitting parameters of each function are given as row vectors. For hierarchical model fitting using activity of individual neurons, the fixed effects are given as row vectors and the random effects given as covariance matrices. The equations of the fitted functions are given in Methods. (PDF) [file pcbi.1011516.s002.pdf]

S1 Table. Parameters of models fitted to neural recording data from the Pavlovian task from [1].

		Inverse square root $[\boldsymbol{m}, \boldsymbol{k}]$	Power $[\boldsymbol{m}, \boldsymbol{k}, \boldsymbol{\pi}]$	Exponential $[\boldsymbol{m}, \boldsymbol{k}, \boldsymbol{\pi}]$
Average		$[0.985, 4.762]$	$[1.677, 4.486, -0.791]$	$[2.148, 5.787, -0.430]$
Hierarchical, correlated parameters	Fixed	$[1.010, 4.697]$	$[1.265, 4.887, -0.750]$	$[2.128, 7.185, -0.680]$
	Random	$\begin{bmatrix} 0.644 & -0.640 \\ -0.640 & 6.363 \end{bmatrix}$	$\begin{bmatrix} 0.124 & -0.718 & 0.027 \\ -0.718 & 6.806 & 0.260 \\ 0.027 & 0.260 & 0.071 \end{bmatrix}$	$\begin{bmatrix} 0.622 & 0.464 & 0.017 \\ 0.464 & 0.953 & 0.216 \\ 0.017 & 0.216 & 0.074 \end{bmatrix}$
Hierarchical, uncorrelated parameters	Fixed	$[0.985, 4.762]$	$[1.094, 4.855, -0.614]$	$[1.969,, 4.783, -0.308]$
	Random	$\begin{bmatrix} 0.538 & 0 \\ 0 & 5.733 \end{bmatrix}$	$\begin{bmatrix} 0.042 & 0 & 0 \\ 0 & 5.610 & 0 \\ 0 & 0 & 0.054 \end{bmatrix}$	$\begin{bmatrix} 0.618 & 0 & 0 \\ 0 & 4.662 & 0 \\ 0 & 0 & 0.019 \end{bmatrix}$

References

1. Lak A, Stauffer WR, Schultz W. Dopamine neurons learn relative chosen value from probabilistic rewards. eLife. 2016;5:e18044. doi:10.7554/eLife.18044.
