# Supplementary material for: Dopamine encoding of novelty facilitates efficient uncertainty-driven exploration
Source: PLoS Comput Biol. 2024 Apr 16;20(4):e1011516. doi: 10.1371/journal.pcbi.1011516 (PMC11051659; doi:10.1371/journal.pcbi.1011516)
Supplement: S2 Table — Mean and variance values across participants of each fitted parameter are given. The definition of models and values of fixed parameters used are given in Table 1. (PDF) [file pcbi.1011516.s003.pdf]

S2 Table. Numerical results from model fitting to behavioural data from [1].

Learning rule	Exploration type	Fitted parameter	Mean	Variance
Kalman filter	Hybrid	γ	1.014	0.225
		θ	0.156	0.049
	Directed	θ	0.902	0.793
		e	3.285	1.837
	Random	γ	1.343	0.274
	Value	e	4.526	1.761
Basal ganglia	Hybrid	α_q	0.728	0.062
		α_s	0.464	0.127
		λ	0.046	0.006
		e	2.039	2.125
	Directed	α_q	0.704	0.070
		α_s	0.467	0.140
		λ	0.033	0.002
		e	2.582	1.904
	Random	α_q	0.750	0.063
		α_s	0.363	0.102
		λ	0.167	0.015
	Value	α_q	0.562	0.083
		e	3.067	2.522

References

1. Gershman SJ. Deconstructing the human algorithms for exploration. Cognition. 2018;173:34–42. doi:10.1016/j.cognition.2017.12.014.
