# Supplementary material for: Dopamine encoding of novelty facilitates efficient uncertainty-driven exploration
Source: PLoS Comput Biol. 2024 Apr 16;20(4):e1011516. doi: 10.1371/journal.pcbi.1011516 (PMC11051659; doi:10.1371/journal.pcbi.1011516)
Supplement: S3 Table — Optimal parameter values (rows) for each of the five tasks (columns) used in simulations. The column titles correspond to the figure panels in Fig 5. Note that OpAL* was only studied in Bernoulli bandit tasks due to its limitation. (PDF) [file pcbi.1011516.s004.pdf]

S3 Table. Optimised parameters for exploration strategies used in simulations.

		(a)	(b)	(c)	(d)	(e)
UCB2	α	0.1166	0.09701	0.1130	0.1543	0.08342
Kalman	λ	2.701	3.034	2.188	0.8334	2.208
OpAL*	α_C	0.025	0.025			0.025
	$\alpha_{G,N}$	0.1	1			0.65
	β	1	1			1
Neural, Fixed LR, $\pi = -0.5$	α_q	0.06279	0.06268	0.06277	0.875	0.06262
	α_s	0.06259	0.06268	0.06244	0.75	0.0625
	λ	0.6262	0.6256	0.6262	0	0.6262
Neural, Fixed LR, $\pi = -0.791$	α_q	0.06265	0.0625	0.0625	0.875	0.0625
	α_s	0.06265	0.0625	0.0625	0.625	0.0625
	λ	0.6249	0.625	0.625	0	0.625
Neural, Dynamic LR, $\pi = -0.5$	$\alpha_{0,q}$	0.06262	0.3749	0.1252	0.75	0.1249
	$\alpha_{0,s}$	0.06238	0.1251	0.1245	1.662e-5	1.300e-4
	λ	0.6261	1.249	1.249	1.271e-3	1.699e-3
Neural, Dynamic LR, $\pi = -0.791$	$\alpha_{0,q}$	0.4646	0.4376	0.375	0.9376	0.3127
	$\alpha_{0,s}$	0.4218	0.4374	0.25	0.9375	0.1874
	λ	5.568	3.123	2.500	0.626	1.874
