# Supplementary material for: Dopamine encoding of novelty facilitates efficient uncertainty-driven exploration
Source: PLoS Comput Biol. 2024 Apr 16;20(4):e1011516. doi: 10.1371/journal.pcbi.1011516 (PMC11051659; doi:10.1371/journal.pcbi.1011516)
Supplement: Attachment — Submitted filename: reviewer_comments.pdf [file pcbi.1011516.s005.pdf]

Response to Reviewer comments

↪ We thank the Editor and the Reviewers for very useful feedback and suggestions. We addressed all of them, as explained in detail below, and we feel that they significantly improved the manuscript.

Reviewer #1

Major comments

Eq 24 is derived under the assumption that samples are IID. I don't think this is mentioned anywhere explicitly? This diverges from the more general assumptions of the Kalman filter, as in Gershman (2018) and subsequent related work.

↪ The strict convergence does require the rewards to be sampled from IID random variables at all trials. We have now addressed this:

> "... *With $S_i[t]$ being the sampling standard deviation and each reward drawn from independent and identically distributed random variables, $\sigma_i[t]$ is an representation estimation of the posterior standard deviation, but since* ..." — *Page 8, Lines 257-259*

The idea of dopamine scaling inversely with novelty is very interesting. I think it might be worth pointing out that this is not by itself sufficient to capture all the behaviors of a Bayesian model like the Kalman filter. In particular, Fan et al. (2023) studied a version of the two-armed bandit where the underlying means diffused over time at different rates. In this case, the sample size could be the same (depending on how often each arms was chosen) but the posteriors would still be different. That paper showed evidence for sensitivity to this form of "epistemic uncertainty" as predicted by the Kalman filter. I'm curious how the authors think about this finding in the context of their model.

↪ This manuscript focuses on a simple and fundamental case of stationary environments. Consequently, a novelty representation by dopamine that monotonically decreases with repeated actions results in low sensitivity to any systemic change in reward structure over time (such as the mean reward diffusion in the task of Fan et al. (2023)). It would be a very interesting direction to extend the model to changing environments. It is possible that to solve such task, dopamine could instead encode a "recency response" similar to that discussed in Xiang and Brown (1998). We have expanded the discussion on non-stationary tasks in the main text:

"… *The Kalman filter as a learning rule is better suited to tackle these non-stationary tasks. A recent study [45] proposed an exploration model that uses Kalman filter in non-stationary bandit tasks, and showed that the Kalman filter, given parameters suitably adjusted to the task environment, can achieve effective learning capable of optimal modulation of exploration in these tasks. The alternative learning rule also based on the GO-NOGO basal ganglia model proposed in [23] uses scaled reward prediction errors, and can approximate a Kalman filter in certain non-stationary tasks without the need to have parameters tuned specifically to the task environment. A meta-learning mechanism that also addresses the generality issue with the Kalman filter was proposed in [46]. Other models with variable learning rate such as the adaptive learning rate models in [47,48] also show significant advantage in their adaptability in non-stationary environments. Beside these normative discussions, recency representation in the brain [49] (which can be considered as novelty representation that discounts observations from a long time ago) could potentially be used to further develop the basal ganglia model and improve the performance of its algorithm in non-stationary tasks, and the resulting algorithm might also serve as a solution to the generality issue of Kalman filter. In sum, there are multiple avenues that can be explored in the future which can help with better understanding of basal ganglia functions in non-stationary reinforcement learning tasks.* …" — *Page 20, Lines 747-765*

I think the major limitation of the paper is that it doesn't present direct evidence for the model's neural prediction in the context of an instrumental task that involves exploration. The only dopamine data analyzed here is from a Pavlovian task, where there are no choices.

↪ We have added an analysis of the choice task from Lak et al. (2016) (S1 Appendix) which gave results in support of our hypothesis, but this analysis is unfortunately less detailed as is based on values read out from printed figures, due to the missing of original data. We added brief reporting of the results in the main text:

"… *The electrophysiological recording data used in this analysis was collected during a Pavlovian learning task, and an experiment with a two-alternative choice task was also performed in the same study [14]. We performed a cursory analysis (S1 Appendix) on the partial dataset obtained from this experiment, and reached similar qualitative conclusions.* …" — *Page 10, Lines 349-353*

My understanding is that λ is constrained to be positive (though this isn't mentioned explicitly in the paper). This assumption, together with Eq. 29, implies that in the regression analyses reported by Gershman (2018) there should be a negative correlation between the coefficients for the relative and total uncertainty terms. To see this, consider what happens if you simulate data from the model for different levels of lambda, but fit the data assuming $\lambda = 1$ (as in Gershman's analysis). Higher values of λ will inflate the relative uncertainty coefficient and deflate the total uncertainty

coefficient. I was curious about this, so I looked at the parameter estimates from Gershman's paper. For experiment 1, the correlation was very close to 0. For experiment 2, the correlation was strongly *positive* ($r = 0.68, p < 0.0001$). I wonder what the authors think about this. More broadly, I'm very interested in whether the authors can pinpoint aspects of their model that make distinctive behavioral predictions relative to previously reported models.

↪ In Eq. 27 (and subsequently Eq. 29), λ is indeed a redundant parameter if η and ν are both free parameters. If fitting this exact action policy to behavioural data, it would make sense to fix the value of λ. In such case, we do not see any reason why η and ν should be negatively correlated since these should independently represent the extent to which each participant engage random and directed exploration. η and ν are not equivalent to the parameters β and γ in Eq. 11 in Gershman (2018), since the term including β includes also the mean difference V. In later analysis in our manuscript, we determined η and ν based on neural data and fitted only λ to the behavioural data, and we did not constrain λ to be positive during this fitting process (the best fitting values turned out to be significantly positive on average). We added an explanation for this in the text:

> "... *With all three parameters λ, ν and η being independent one is redundant. We address this later with additional constraint based on experimental results.* ..."
> — *Page 9, Lines 286-288*

From a purely psychobehavioural perspective, we think that the main difference of the model compared with the hybrid model in Gershman (2018) is actually in the learning rule rather than choice policy, but under most task conditions there still would not be a distinctive qualitative difference, as has been demonstrated to an extent by both the fitting of behavioural models to the data and the performance comparison in simulations. Novel predictions are mostly made on the results of intervention of the dopaminergic pathway. These are detailed in Discussion (Lines 676 to 775).

Minor comments

I'm not sure why there's a comma in $V_{\mathrm{UCB},i}[t]$ and $V_{\mathrm{Thompson},i}[t]$.

↪ We are trying to use two sets of subscripts here to denote exploration types (this is unique to the decision value utility variables) and action indices. We think this is necessary for rigorousness.

Fig 4 caption: "trail-by-trial" should be "trial-by-trial".

↪ This has now been corrected.

What do the error bars show in Fig 4? Please note this in the caption. I suspect that these are between-subject error bars, when what you want here is within-subject error bars that reflect the paired comparisons.

↪ The figure in the original draft did in fact show between-subject error bars. We have now changed these to within-subject error bars and noted this in the caption.

Line 587: " . . . Gershman and Tzovaras (2018) found that stronger striatal dopamine transmission reduced the effect of relative uncertainty on directed exploration." I would word this differently, since that study looked at single nucleotide polymorphisms, not dopamine transmission. There is a connection but it is indirect.

↪ We have changed the wording to make this argument more rigorous:

> *". . . in [38] the correlation between behavioural traits in exploration tasks and single nucleotide polymorphisms of dopaminergic genes was studied, and results show that variation in a gene linked to striatal dopamine predicts the effect strength of relative uncertainty differently than our current model does, in that the genotype associated with higher level of striatal dopamine transmission correlates with reduced effect of relative uncertainty on directed exploration. . . . "* — *Page 17, Lines 587-592*

Gershman (2018) reported two experiments. Which ones did you fit?

↪ We only fitted data from Experiment 2 in Gershman (2018), as we did not want to add in an ad hoc element in our behavioural model to account for the prior knowledge about the deterministic option in Experiment 1. We have now made this explicit in the main text:

> *". . . We used data from one of two experimental setups in [12] (Experiment 2), where both options of the two-armed bandit were associated with stochastic rewards. . . . "* — *Page 12, Lines 387-388*

Reviewer #2

Multiple roles of dopamine. This paper proposes several different effects of dopamine in different timings.

1. Plasticity D1 and D2 MSNs by Eqs. 9 and 10.
2. Modulation of thalamic activity by direct/indirect pathways by Eq. 22.

While effect on plasticity is supposed to be for the timing after action selection, modulation of thalamic activity is for the timing of action selection. Experimental data of Lak et al. (2016) shows novelty-dependent responses within 0.1 to 0.2 s from cue onset. Action selection, however, may not happen within that time frame. Subjects may choose an action with a longer delay, or even spontaneously without any cue. In the choice task of Lak et al. (2016), the saccade reaction time was about 50 ms shorter in early exploratory trials, but it was around 600 to 650 ms. The dopamine response had two peaks; first at 0.1 - 0.2 s which was novelty dependent and second at 0.4 - 0.65 s, which was value coding. It appears that the second component is more relevant for the action choice. This link between the very early novelty-dependent dopamine and action selection that can happen later should be better explained.

↪ This apparent discrepancy in timing could be explained by the time required for downstream computing processes before decision is formed. According to the model, the dopaminergic novelty signals occurring at 0.1 - 0.2 s serve to rapidly facilitate/depress the corticostriatal projections, and subsequently the activation of MSNs is modulated by these effects to produce value signals that combine estimated mean reward, reward variability and novelty. It is also reasonable that the value coding signals at 0.4 - 0.65 s tend to immediately precede action onset, as at this point the decision has been formed internally and expectations of the outcome is encoded by dopamine (this is well documented). We have added this discussion to the main text:

> *"... During this task, animals typically made saccade actions between 0.6 to 0.65 s after cue onset, which was better aligned with the reward predicting phase of dopaminergic activity rather than novelty encoding phase. According to our model, the novelty signal is involved in action selection, while the subsequent reward-correlated signal is used for learning. It is possible that the delay between novelty signal and action is the time required to integrate the signals for different actions and form decision and action; during this time, an internal prediction of the action outcome is also formed and encoded by the reward-predicting activity. ..." — Page 17, Lines 619-626*

Meaning of i: In the mathematical formulation of Eq 22 and onwards, i should be the index of neural population coding a certain action, and the dopamine activity D_i is also supposed to be different for different cues or actions.

↪ This is correct. We have now made this explicit when introducing the nomenclature:

> *"... Later in this article, we also use the same subscript i to denote activities of neuronal populations that encode latent variables associated with the action of choosing a certain arm. ..." — Page 3, Lines 95-97*

An important question is whether dopamine neurons can simultaneously carry different novelty signals for different cues or actions, given the wide-spread nature of dopamine projection, e.g., in Matsuda et al. (2009). It might be that dopamine neurons respond to the most novel cue, as in Lak et al. (2016), but a question is how that can affect the value of the most novel cue.

↪ This is indeed a very important question for future studies. In short, we believe that the existing knowledge on dopaminergic neuron arborisation does not conclusively support or negate our assumption of simultaneous representation of independent novelty signals, and we suggested potential methods of further testing through experiment. We have added more extensive discussion on this particular issue:

> "... *An important assumption made by our model is that the novelty level of each individual available action can be independently represented. We unfortunately do not have sufficient data to verify this in the current study. Based on previous investigation on the morphology of axonal projection of dopaminergic neurons [42], each dopaminergic neuron can project to thousands of neurons in the striatum, whereas the number of dopaminergic neurons that can influence each striatal neuron is lower by orders of magnitude. This shows that the level specificity of dopaminergic projection is not particularly strong, but should still theoretically be sufficient to support the mechanism proposed by our model.* ..." — *Page 17, Lines 627-635*

Performance in simulation: Fig 5 presents the simulation results for a virtual experiment with 10 actions over 5000 trials, but it is more helpful if such a simulation is done for experiments with animal or human data. Most importantly, for the dataset of Gershman (2018), in addition to model comparison by BIC, it is interesting to compare the simulated performances of different models with the fitted parameters. It is also interesting to do the same for the choice task of Lak et al. (2016).

↪ The reason for using a difficult task and simulating over long blocks of trials is to better differentiate the algorithms by their performance. The simulations are also tangential to the analyses of experimental data in their purpose, so we did not expect much added value in using the same tasks in simulation as those used in experiments. Nevertheless, we did simulate the experiment from Gershman (2018) that we analysed. We have now added this work to Results (see Fig 5D). It is difficult to separate the performance of most of the algorithms tested in this task.

Detailed comments

In line 19, the description of the multi-armed bandit task can be more concrete. In line 90, the authors describe this task again. It might be better to put two parts together.

↪ We are leaning towards keeping the current structure, as we do not wish to bring in the nomenclature earlier in the introduction, but a somewhat detailed description of the task is important for context given the computational nature of this work. We have added a sentence to point to the later mathematical description:

> "... *We formalise the mathematical description of the task later in Introduction.* ..." — *Page 2, Lines 30-31*

In line 32, the authors wrote, "If an agent follows a greedy strategy that does not involve any exploration at all and always prefers the optimal action according to current knowledge, they would simply play each arm exactly once at the beginning of each block of trials." If the agent also plays greedily without any prior knowledge, shouldn't it stick to the option that gives the first reward?

↪ This is a valid definition for a fully greedy strategy if the rewards are binary, but it does not generalise if the rewards are sampled from continuous distributions. We think that sampling each option once and choosing the most rewarding one in all following trials is still a valid interpretation, and this also generalises to tasks with continuous reward distributions.

In Section , it would be clearer to state this study assumes the environment is stationary before further discussion.

↪ We mentioned stationary environment earlier in the introduction, and have now added a sentence reiterating this in Section :

> "... *Another caveat of this representation of posterior uncertainty is that it is only valid for stationary environments where the reward distributions do not change over time, which the scope of this study is confined to. We come back to this in Discussion.* ..." — *Page 8, Lines 263-266*

For Eq. 25, the meaning of η and ν should be explained.

↪ We have now defined these parameters after the equation:

> "... *ν and η are constant parameters which scale the effect of novelty on the average and variability of dopamine response, respectively.* ..." — *Page 9, Lines 276-277*

After model fitting, the authors refine the DA response from Eqs 24 to 35 and the value signal from 27 to 36. Is it possible to explicitly describe the choice probability given by this refined model as Eq 29 and discuss whether the refined version shares those essential properties as well?

↪ We have now added the choice probability according to the refined model, along with discussion on the quality of the new choice strategy in comparison with the ideal version (Lines 364 to 370).

In Section , the authors explain that "the decrease in BIC from better fitting is outweighed by the increase from additional penalty for extra parameters." To support this statement, authors should also report the fitting result before penalization by parameters, e.g. average (log) likelihood.

↪ We have now added the comparison of AIC values (minimal parameter penalty) alongside the BIC comparison in Fig 4, and added discussion about the findings:

> "… *This is supported by the AIC values (which include a smaller penalty for number of parameters in this case) shown in Fig 4A, which suggests significantly better fitting of all basal ganglia models compared with their Kalman filter counterparts ($p < 0.05$, $p < 0.0001$, $p < 0.001$, $p < 0.0001$ respectively for the four exploration types in order, two-tailed t-tests).* …" — *Page 13, Lines 432-436*

For performance comparison in Section , in addition to the regret, the actual performance and the frequency of exploratory choices may also be better reported.

↪ We added an explanation for choosing regret as the performance indicator:

> "… *Importantly, for a given choice, regret is a deterministic quantity while reward may be stochastic, hence regret is a more consistent and less noisy variable in simulations than the reward.* …" — *Page 14, Lines 465-467*

The authors found that the neural strategies with fixed learning rates were worse than normative strategies and then used the neural strategies with varying learning rates. It is not surprising that a model with time-varying parameters has better performance. To show the efficiency of neural strategies, it is better to introduce other models with time-varying parameters and compare their performance.

↪ The Kalman filter algorithm also involves a varying learning rate (one that is arguably more sophisticated than the decay-with-time version), and as this algorithm also has a similar choice policy, the performances are understandably similar. Some of the classic strategies from Auer et al. (2002) also effectively have variable learning rates as they use the exact historic mean reward values. We have now also added another recently proposed algorithm (Jaskir and Frank, 2023) (see Fig 5) that involves both dynamic learning rates and modulated exploration. The reason we are not including a simple ε-greedy type algorithm with dynamic learning rate is that we want to keep all algorithms in the comparison fully deterministic in their choice policy (i.e. given the current learned latent variables, there is no randomness in the decision), which ε-greedy cannot achieve.

In Section , authors mentioned the risk-seeking and novelty-seeking behavior, which appeared several times before. Authors should make a clear difference between risk and novelty in relevant discussion.

↪ We first mentioned "risk seeking" in Line 67, and gave a definition of this term:

> "… *Specifically, higher dopamine level should result in a stronger preference for more risky actions with more variable outcomes.* …" — *Page 3, Lines 67-69*

We have now reiterated this in Line 235:

> "... *(i.e. increased tendency of choosing actions with more variable outcomes)* ..." — *Page 8, Lines 235-235*

We used the phrase "novelty seeking" in Line 584, and we have now added a parenthesis after this:

> "... *(preference towards the less-chosen actions in the context of multi-armed bandit)* ..." — *Page 16, Lines 584-585*

The "uncertainty" we discuss in this work is essentially a combination of novelty and risk by our definitions (in the context of a stationary task).

Reviewer #3

There is a slight ambiguity in the adopted definition of exploration: "In this study, we generalise from real-world scenarios and define exploration to be any behaviour by a learning agent that favours actions which are sub-optimal in terms of their expected rewards according to the current best knowledge, and exploitation as behaviour that chooses the optimal action with highest expected reward." It is not clear if the "current best knowledge" is from the point of view of the agent or from the point of view of an ideal observer, which would lead to different labeling of exploratory trials.

↪ Here "current best knowledge" is from the point of view of the agent. We have clarified this in text (Line 10).

Modulated exploration does not necessarily mean directed exploration. See for instance Velentzas et al. 2017 RLDM for a comparison of classical UCB methods with an algorithm instantiating active tuning of random exploration rate. Importantly, such a dynamic random exploration rate enables fast adaptation to non-stationarity, similar to the dynamic learning rate models discussed lines 756-758.

↪ We are indeed making a distinction between modulated exploration and directed exploration in our work. In our view, any algorithm that uses learned latent representations to form exploratory actions qualifies for modulated exploration. As such, the directed, random and hybrid exploration we refer to in this word following the naming conventions of Gershman (2018) are all considered modulated exploration. Unfortunately, our model in this study does not generalise well to non-stationary tasks due to the way the posterior uncertainty is represented by the learning mechanism. The algorithm of Velentzas et al. (2017) is interesting in its use of a meta-learning component to modulate random exploration. We have now added this work to Discussion:

> "... *A meta-learning mechanism that also addresses the generality issue with the Kalman filter was proposed in [46].* ..." — *Page 20, Lines 755-756*

We also expanded on other potential ways of extending our model to better

adapt to non-stationary tasks following this.

Lines 65-67 “Based on this assumption, tonic dopamine level in the striatum can influence the overall level of risk seeking in behaviour because of the opposite effects dopamine has on D1 and D2 neurons.” This is precisely the hypothesis put forward by Humphries et al. (2012). However, Humphries and colleagues made the assumption that the role of tonic dopamine is opposite to the one presented here that “higher dopamine level should result in a stronger preference for more risky actions with more variable outcomes.” In Humphries et al. (2012) such a positive relation between dopamine and exploration could be obtained only with specific parameters determining the strength of basal ganglia control over target structures (blue area in their Fig 6A), which they argued could correspond to specific basal ganglia territories. However, they assumed that the most common effect of tonic dopamine in the basal ganglia on exploration should be negative: more dopamine should produce more exploitation. This topic is partially discussed in the present paper. Nevertheless, some further discussion would be useful. Can the difference be due to different connectivity patterns in different basal ganglia territories? Is it due to different species (rodents, primates)? Is it due to different mechanisms relying on tonic versus transient versus phasic dopamine signals? How many papers found experimental evidence in each different species in favor of positive versus negative influence of dopamine on exploration?

↪ The model of Humphries et al. (2012) is obviously a more detailed account of the basal ganglia dynamics compared to our trial-by-trial model. It is entirely possible that these details lost in abstraction can in fact lead to a different qualitative prediction. As of experimental evidence, it is frankly unclear to us how to reconcile some of the seemingly contradicting observations. The effect of tonic dopamine levels in different brain areas on exploration appears to be very complex and multi-dimensional, as risk preference, overall choice stochasticity and modulated random exploration can all apparently be influenced independently of each other. Although our analysis in this work is fundamentally based on the model that predicts a positive correlation between risk seeking and tonic dopamine level, it is very much focused on the function of phasic dopaminergic activity, and therefore we did not dive deep into the discussion on the effect of tonic level. Judging from literature, we do not believe there is a significant effect coming from the species used for experiment. We are keen to further look into these important issues in our future work.

Please cite Collins and Frank (2014) when presenting the learning rules of the GO and NOGO pathways (Line 182).

↪ We have added the requested citation:

> “. . . *This follows the naming convention of [27], who first proposed the OpAL model which is closely connected to the model we describe in this work.* . . . ” *— Page 6, Lines 179-180*

Line 194 The interval for alpha should be written $[0; 1]$ rather than $(0, 1)$.

↪ We have now changed it to the closed interval.

Line 250 "the most effective" rather than "the more effective"?

↪ The intended comparison was between the posterior uncertainty (σ) and reward standard deviation (S), but context was insufficient. We have now changed the wording:

> "... *However, as mentioned in Introduction, the posterior uncertainty of mean reward $\sigma_i[t]$ (rather than reward variability) is the normatively optimal modulator for exploration that can facilitate an effective algorithm throughout the task.* ..." — *Page 8, Lines 250-253*

I like very much the elegant way of formalizing dopamine novelty signals as inversely correlated to the sample size in a parameterized way that permits to reduce the model to normative exploration strategies (UCB and Thompson sampling) when setting the corresponding parameters to 0. Nevertheless, this assumes that tonic or transient dopamine encodes novelty, and that dopamine in this model in general has nothing to do with reward nor reward prediction errors. In other words, dopamine only affects exploration here, and an arm i's mean reward value Q_i and mean deviation of reward S_i are updated by a delta signal which has nothing to do with dopamine. These aspects should be discussed here. Aren't dopamine responses to novelty a component of dopamine phasic activity? Moreover, could the author elaborate on the link or interplay between dopamine phasic response used for reinforcement learning and dopamine tonic or transient response used for action selection regulation? Is McClure et al. (2003) model proposing a relevant mechanism for integrating phasic and tonic dopamine in the present framework?

↪ We wish to clarify that we still believe dopamine takes part in reinforcement learning belief updates in our model (Lines 545 to 552). We do not have the recording data from after reward onset to perform a formal analysis, but the late-phase reward predicting signals of recorded neurons suggests that there is potential for dopaminergic neurons to perform these two separate functions. Therefore, we think that the McClure et al. model is not very relevant to the current work, as it only uses dopaminergic signal for learning.

Fig 4 Caption, trial-by-trial instead of trail-by-trial. Two closing parentheses are needed after "see Methods for details".

↪ These have now been corrected.

In the fit to behavioural data, the basal ganglia model performs similarly (in terms of BIC) to Gershman's Kalman filter, while it has more parameters and is thus probably over-penalized by the BIC. I recommend either testing another criterion, e.g. corrected AIC (Cavanaugh, 1997) or integrated BIC (Wittmann, 2020), or reducing even further the number of optimized parameters, by making additional assumptions, e.g. $\alpha_q = \alpha_s$.

↪ Another reviewer made similar suggestion to add comparison of raw likelihoods. We added the simple standard AIC alongside BIC as a compromise (Fig 4). We added the following discussion on the findings:

"... *This is supported by the AIC values (which include a smaller penalty for number of parameters in this case) shown in Fig 4A, which suggests significantly better fitting of all basal ganglia models compared with their Kalman filter counterparts ($p < 0.05$, $p < 0.0001$, $p < 0.001$, $p < 0.0001$ respectively for the four exploration types in order, two-tailed t-tests).* ..."
— *Page 13, Lines 432-436*

Could the authors add a table showing the value of the optimised parameters for each experiment (electrophysiology, human behaviour, simulations)?

↪ For the electrophysiology experiment, we fitted functions using both the average activity of the recorded neurons and activity of the individual neurons with hierarchical model fitting. From fitting using individual neurons, there are hundreds of resulting parameters (as there is one set of optimal parameters for each participant), so these are not tabulated (we plan to make the code available, and the repository we plan to publish will include data files containing all model fitting results), but we have now included the group-level mean and covariance of each model resulting from hierarchical fitting in Supporting Information (**S1 Table**). For the fitting to average activity, we have now also added the best fitting parameters to the same table.

For the behavioural data, we fitted parameters to data from individual participants only, so again there is a vast quantity of resulting parameters. These will also be made available with the code. We have included the mean and variance values of the fitted parameters in **S2 Table**.

For the simulations, we have now added a table of optimised parameters in **S3 Table**.

Could the authors also indicate the reward mean and variance associated to each arm in the human behavioural experiment?

↪ We have now clarified on this:

"... *The mean rewards themselves were drawn from a zero mean Gaussian distribution ($\mathcal{N}(0, 100)$) for each block, and the reward variance of each arm was fixed at 10.* ..."
— *Page 12, Lines 391-393*

In fitting the different models to human behaviour, the authors did not observe a significant difference between the fixed learning rate neural models and the Kalman filter model, unlike in the bandit tasks simulations. The authors interpreted this different to be likely due to the behavioural experiments involving much shorter blocks of trials compared with the simulations. However, this suggests than within each 10-trial block of the human experiment, the neural models are not as good as the Kalman filter model, but rather as bad as it. Again, it would be useful to see the optimised parameters of these models in each experiment, as well as the learning curves in the human experiment, to see how far from optimality the models are.

↪ We wish to emphasise that the analysis of behavioural data is to establish that, with the different learning rule derived from the basal ganglia model, the hybrid exploration behavioural model still has the greatest explaining power of human behaviour, just like the Kalman filter equivalent – we believe we have demonstrated this sufficiently. The fact that the two different learning rules result in similar explanation powers of behaviour is down to the difference between mean rewards of the two arms being large relative to the reward variances, so any learning rule with a high enough initial learning rate can achieve similar learning results. The purpose of the simulations, on the other hand, is to gauge the theoretical performance of each algorithm in different bandit tasks. The best performing algorithm does not necessarily correspond to the best-fitting behavioural model. Furthermore, we have now added the same task in the behavioural experiment analysed to our simulations (see Fig 5 D), and showed that the fixed learning rate basal ganglia algorithm has much more comparable performance to the Kalman filter than in the other tasks, showing that the difference in learning rule is also less critical to performance in such a task.

The paper only addresses stationary tasks, while non-stationarity is a key test bed for exploration strategies and novelty-seeking. Could the authors also compare their neural model and the Kalman filter model with classical exploration models in non-stationary tasks?

↪ The classic UCB algorithms as well as the neural model algorithm are not suited for non-stationary tasks (we would expect the Kalman filter model to significantly outperform these). We suggest to further develop neural model to account for non-stationary tasks in future work before simulating these non-stationary tasks. We have now expanded the discussion on this particular matter:

"... *The Kalman filter as a learning rule is better suited to tackle these non-stationary tasks. A recent study [45] proposed an exploration model that uses Kalman filter in non-stationary bandit tasks, and showed that the Kalman filter, given parameters suitably adjusted to the task environment, can achieve effective learning capable of optimal modulation of exploration in these tasks. The alternative learning rule also based on the GO-NOGO basal ganglia model proposed in [23] uses scaled reward prediction errors, and can approximate a Kalman filter in certain non-stationary tasks without the need to have parameters tuned specifically to the task environment. A meta-learning mechanism that also addresses the generality issue with the Kalman filter was proposed in [46]. Other models with variable learning rate such as the adaptive learning rate models in [47, 48] also show significant advantage in their adaptability in non-stationary environments. Beside these normative discussions, recency representation in the brain [49] (which can be considered as novelty representation that discounts observations from a long time ago) could potentially be used to further develop the basal ganglia model and improve the performance of its algorithm in non-stationary tasks, and the resulting algorithm might also serve as a solution to the generality issue of Kalman filter. In sum, there are multiple avenues that can be explored in the future which can help with better understanding of basal ganglia functions in non-stationary reinforcement learning tasks.* ..." — *Page 20, Lines 747-765*
